# Clinical Applications of High Resolution In-Vivo Retinal Imaging

**DOI:** 10.1155/2013/312974

**Published:** 2013-02-27

**Authors:** Stacey S. Choi, Ann E. Elsner, Robert J. Zawadzki, Brian Vohnsen

**Affiliations:** ^1^Department of Vision Science, New England College of Optometry, Boston, MA 02115, USA; ^2^School of Optometry, Indiana University, Bloomington, IN 47405-3680, USA; ^3^Department of Ophthalmology and Vision Science, University of California, Davis, Sacramento, CA 95817, USA; ^4^School of Physics, University College Dublin, Dublin 4, Ireland

The last two decades have witnessed major advancements in retinal imaging technologies thanks, in no small part, to improved light sources and detectors, adaptive optics, and high-speed computers. New research grade systems are continuously improving [1] and some have obtained a degree of user friendliness and robustness that have facilitated their transition to clinical use. Ultrahigh resolution commercial scanning laser ophthalmoscopy (SLO) and optical coherence tomography (OCT) systems are becoming commonplace in modern ophthalmic clinics where they provide often unprecedented image quality with resolving powers down to the level of single retinal cells in the living eye. Such richness in detail provides challenges and possible paradigm shifts, but already an enormous impact on improved diagnostics has been realized as we continue to work towards the ultimate goal of achieving healthy vision throughout life. 

The six papers included in this special issue cover some of the recent advances on the clinical applications of high resolution, in vivo retinal imaging using an array of leading imaging technologies and in particular spectral-domain- (SD-) OCT. While SD-OCT has been widely accepted for retinal imaging little more than a decade ago, it has rapidly become the method of choice for fast ultrahigh resolution cross-sectional imaging that produces histology-like results, as well as 3D imaging of structures within the eye [2]. With broadband spectral illumination, axial resolution on the order of 2-3 microns [3] has become possible, which is close to the transverse resolution limit of about 2 microns achievable with adaptive optics and dilated pupil [4]. 

One of the chief concerns in the ageing population is age-related macular degeneration (AMD), as it can have a detrimental impact on the life quality of affected individuals. A number of risk factors for AMD development have been identified. The majority of cases present as dry AMD, in which the appearance of deposits hidden beneath the retina, called drusen, provides an early sign of the disease. The less common wet AMD is typically more severe, with development of new vessels that leak and lead to the formation of scars. It is the wet AMD that leads more often to a rapid and debilitating loss of central vision. B. Wolff et al. show imaging results of outer retinal tubulations that can form as a consequence of wet AMD. *En face*, ultrahigh resolution SD-OCT scans in the transverse direction show a network of scarring in what the authors term as “pseudodendritic.” 

The neurodegenerative Parkinson's disease (PD) is another concern in the ageing population that relates to protein aggregation of amyloid fibers in the brain but also has implications for the eye and vision. E. M. Shrier et al. report on SD-OCT scans of foveal thickness in PD patients in relation to a thinning of the nerve fiber layer. To differentiate between retinal layers, they focused their study on the foveal pit and found that about half of the patients had substantial intraocular asymmetry of the slope of the foveal pit slightly off-axis from the foveola. Thus, these differences may potentially be used as indicators of PD.

The review article by Y. Barak et al. discusses the ageing process of the vitreoretinal interface, vitreous shrinkage, and associated pathologies visualized with SD-OCT. Examples described include posterior vitreous detachment and the development of floaters, vitreomacular traction causing retinal swelling and reduced vision, and macular holes that may develop into full thickness macular holes requiring surgical intervention to prevent vision loss. The paper also discusses future perspectives including the exciting possibility of intraoperative SD-OCT that will allow real-time monitoring of changes taking place during macular surgery. 

Two commercial SD-OCT and time-domain OCT instruments are compared in a study of retinal nerve fiber layer thickness in 132 healthy subjects by I. Pinilla et al. Both techniques give similar results of 95 to 98 micron thickness, though the accuracy is found to be higher with SD-OCT in agreement with also other studies. Retinal nerve fiber layer thickness is related to early diagnosis of glaucoma, in which accurate determination of thinning is of utmost importance. 

A comparison of SD-OCT and time-domain OCT instruments is again central in the work by A. P. Lange et al. related to retinal nerve fiber layer thickness in the eyes of multiple sclerosis (MS) patients suffering from optic neuritis. A systematic 2-micron difference in measured thickness is reported between the two instruments, similar to the conclusion reached by I. Pinilla et al. This stresses the importance of specifying the device used in any comparative study. MS patients have a thinner retinal nerve fiber layer thickness in comparison to a control group, and in particular MS patients with a history of optic neuritis show a substantial reduction in the retinal nerve fiber layer thickness. 

The final paper by S. Kawaguchi et al. deals with ocular blood flow, carotid revascularization surgery, and chronic ocular ischemic syndrome. Color-coded Doppler flow imaging before and after revascularization surgery confirmed an increased flow velocity, and visual acuity increased in 60% of the patients. 

All six papers in this special issue have undergone a rigorous peer review process, and we are thankful to the referees for their work to meet the quality requirements of the accepted papers to ensure that it conforms to the standards of the Journal of Ophthalmology. We hope that the readers will find the papers of interest as they show essential parts of the spectrum of clinical work already being done with ultrahigh and high resolution retinal imaging techniques and that they may inspire future clinical work. Given the relatively novelty of ultrahigh resolution retinal imaging technologies, the future looks promising for much improved diagnostic imaging as the limits of the methods are removed and their clinical usage continues to grow.

*Stacey S. Choi* *Stacey S. Choi* *Ann E. Elsner* *Ann E. Elsner* *Robert J. Zawadzki* *Robert J. Zawadzki* *Brian Vohnsen* *Brian Vohnsen*
